# Patients with community-acquired bacteremia of unknown origin: clinical characteristics and usefulness of microbiological results for therapeutic issues: a single-center cohort study

**DOI:** 10.1186/s12941-017-0214-0

**Published:** 2017-05-19

**Authors:** Johan Courjon, Elisa Demonchy, Nicolas Degand, Karine Risso, Raymond Ruimy, Pierre-Marie Roger

**Affiliations:** 1 Infectious Diseases Department, Hôpital Archet 1, Nice Academic Hospital, Infectiologie 151 Route de St Antoine de Ginestière, 06200 Nice, France; 2 Université Côte d’Azur, Nice, France; 3 Department of Bacteriology, Archet 2 Hospital, Nice Academic Hospital, Nice, France; 4 INSERM U1065 (C3M), Bacterial Toxins in Host Pathogen Interactions, C3M, Archimed, Nice, France

**Keywords:** Bacteremia, Empirical antibiotic treatment, Severe sepsis, Bacteremia of unknown origin, Antimicrobial resistance, Primary bacteremia

## Abstract

Bacteremia of unknown origin (BUO) are associated with increased mortality compared to those with identified sources. Microbiological data of those patients could help to characterize an appropriate empirical antibiotic treatment before bloodcultures results are available during sepsis of unknown origin. Based on the dashboard of our ward that prospectively records several parameters from each hospitalization, we report 101 community-acquired BUO selected among 1989 bacteremic patients from July 2005 to April 2016, BUO being defined by the absence of clinical and paraclinical infectious focus and no other microbiological samples retrieving the bacteria isolated from blood cultures. The in-hospital mortality rate was 9%. We retrospectively tested two antibiotic associations: amoxicillin–clavulanic acid + gentamicin (AMC/GM) and 3rd generation cephalosporin + gentamicin (3GC/GM) considered as active if the causative bacteria was susceptible to at least one of the two drugs. The mean age was 71 years with 67% of male, 31 (31%) were immunocompromised and 52 (51%) had severe sepsis. Eleven patients had polymicrobial infections. The leading bacterial species involved were *Escherichia coli* 25/115 (22%), group D *Streptococci* 12/115 (10%), viridans *Streptococci* 12/115 (10%) and *Staphylococcus aureus* 11/115 (9%). AMC/GM displayed a higher rate of effectiveness compared to 3GC/GM: 100/101 (99%) vs 94/101 (93%) (p = 0.04): one *Enterococcus faecium* strain impaired the first association, *Bacteroides* spp. and *Enterococcus* spp. the second. In case of community-acquired sepsis of unknown origin, AMC + GM should be considered.

## Background

Bacteremia is defined by the presence of viable bacterial agent in the bloodstream and is diagnosed in daily clinical practice with the use of blood cultures. The annual incidence of community-onset bacteremia is evaluated between 40 and 154/100000 [1]. The range of mortality rates is extremely large depending on the occurrence of septic shock, antimicrobial resistance, age, comorbidities and community or healthcare-acquired infections. Bacteremia corresponds to bacterial dissemination through the bloodstream from an initial infectious focus, among which urinary sepsis, pneumonia and intravascular catheter-related infection are the most frequent [2, 3]. Nevertheless, even with an exhaustive diagnostic procedure, this portal of entry sometimes remains unknown.

The definitions of BUO vary significantly according to the different authors. In a study of Vallès et al. [4] the absence of a distal source documented is sufficient, whether other authors use a composite criterion [5] combining non-contributing physical exam, lack of microbiological investigations identifying the same bacteria as in bloodcultures and normal radiological exams.

BUO is associated with significant morbidity compared to bacteremic patients with an identified infectious focus [6]. Even in intensive care units (ICU) where huge risk factors as septic shock or ICU scoring systems often overshadow other clinical determinants, BUO has been associated with inappropriate antibiotic treatment [4] and poor outcome [7].

When empirical antibiotic treatment is required, the decision process usually includes the infection focus identified by physical exam or the first radiological results. During BUO, presenting as sepsis of unknown origin, these data are missing and could favour inappropriate antibiotic administration, which is known to increase mortality [8]. Nowadays, the challenge for empirical antibiotic prescription is to combine appropriate antibiotic use and to consider the risk of antimicrobial resistance in the community setting especially for 3rd generation cephalosporin resistant *Enterobacteriaceae* [9].

While studies focusing on BUO are scarce we present herein a cohort of 101 community-acquired BUO seen in our department. This study aims to describe clinical presentation and the main features of patients involved. On the basis of the microbiological results of our cohort we have tested the efficiency of two antibiotic associations which both include a beta-lactam with an aminoglycoside. The main objective is the characterization of a treatment that could be used, by extension, during sepsis of unknown origin.

## Patients and method

### Patients’ selection and ethical approval

This retrospective cohort-study was conducted from July 2005 to April 2016 in the 34-bed infectious diseases department at Nice University Hospital (France). The medical dashboard of our ward records prospectively 28 characteristics of each hospitalization including hospitalization motive, final diagnosis, comorbidities, microbiological data including blood culture and all antibiotics prescribed [10]. Patients are classified regarding the site of infection; in case of bacteremia without any organ infection detected they are included in the BUO group. The dashboard classifies infective endocarditis or spondylodiscitis in other groups. In France no ethical approval is required for non-interventional study. The medical dashboard of the Infectious Diseases Department of Nice University Hospital is authorized by the French National Commission on Informatics and Liberty (Number of Registration: 1430722). A signed consent form is used in our hospital for each patient in order to enable the use of the clinical data recorded during current care for medical research.

### Definition of community-acquired BUO used for the study

The origin of the bacteremia was considered as unknown when clinical and paraclinical data failed to identify any infectious focus and when no other microbiological samples retrieved the bacteria isolated from blood cultures.

### Inclusion and non-inclusion criteria

All patients classified in the BUO group were selected. After reviewing all the medical files, patients meeting the definition of community-acquired BUO used for the study were included.

Patients with coagulase-negative *Staphylococci* bacteremia and fungemia were excluded. Because of the difficulty to strictly rule out a cutaneous primary infection in case of chronic ulcers, pressure ulcers or in intravenous drug users, those patients were excluded. Health-care associated infections were also excluded.

### Variable of interest

Symptoms leading to the emergency department before hospitalization were collected from the emergency department medical record. Fever was defined as a body temperature ≥38.3 °C. Inappropriate empirical treatment referred to an antibiotic administered before the blood cultures results, to which at least one bacteria isolate was resistant. The number of positive blood cultures bottles for each patient was recorded.

Results of the following exams were collected in the medical record: level of C-reactive protein (CRP) and/or procalcitonin (PCT) at day one, thoracic and abdominal CT-Scan, transthoracic and trans oesophageal echocardiography (TTE and TOE) and colonoscopy.

Duration of follow-up was determined by the date of the last visit in our hospital. Follow-up phone call was performed for the patients who did not visit our hospital after the treatment of the BUO in order to identify any recurrence of BUO. Unfavourable outcome included ICU transfer or death during in-hospital care.

### Antibiotic combination evaluation

Based on the antimicrobial susceptibility testing, we retrospectively evaluated the efficiency of two antibiotic combinations for the treatment of BUO: amoxicillin–clavulanic acid + gentamicin (AMC/GM) and 3rd generation cephalosporin (cefotaxime or ceftriaxone) + gentamicin (3GC/GM). These associations were considered as active if the causative bacteria were susceptible to at least one of the two drugs.

### Severe sepsis and organ dysfunction

Sever sepsis was defined by the *Surviving Sepsis Campaign*: systolic blood pressure <90 mmHg or mean arterial pressure <70 mmHg, lactate above upper laboratory limits, PaO_2_ < 60 mmHg or pulse oximetry <90% in air, creatinine >176.8 μmol/L, platelet count <100 G/L or INR > 1.5, hyperbilirubinemia >70 μmol/L and also altered mental status.

### Microbiological findings

Blood samples were collected in a set of an aerobic and an anaerobic bottles which were incubated in a BacT/ALERT 3D (bioMérieux, Marcy l’étoile, France) automated blood culture system for 5 days. Bottles that showed a positive signal in the BacT/ALERT 3D system were routinely subjected to Gram staining and subcultured at least on blood agar plates and upon results of Gram on Drigalski agar or on chocolate agar. Colonies were identified using the API system (bioMérieux) and, since 2013, MALDI-TOF MS Microflex LT (Bruker Daltonics GmbH, Bremen, Germany) according to the manufacturer’s recommendation. Antimicrobial susceptibility testing was performed in accordance with the EUCAST disk diffusion test methodology, as recommended [11].

### Statistical analysis

The analysis was performed using StatView^®^F-4.5. The relationship between variables were assessed with the Chi2 test for categorical variables; Fisher’s exact test was used for number of variables <5. Continuous variables were compared using the Mann–Whitney non-parametric test. Logistic regression was used for the multivariate analysis of risk factors associated to in-hospital mortality or ICU transfer during BUO and results are presented as adjusted odds ratios (AORs) with their 95% confidence intervals (CIs). Variables were selected as candidates for the multivariate analysis on the basis of the level of significance of the univariate (p < 0.1). Models were built up sequentially, starting with the variable most strongly associated with the outcome and continuing until no other variable reached significance or altered the odds ratios of variables already in the model. When the final model was reached, each variable was dropped in turn to assess its effect.

## Results

**Fig. 1:**
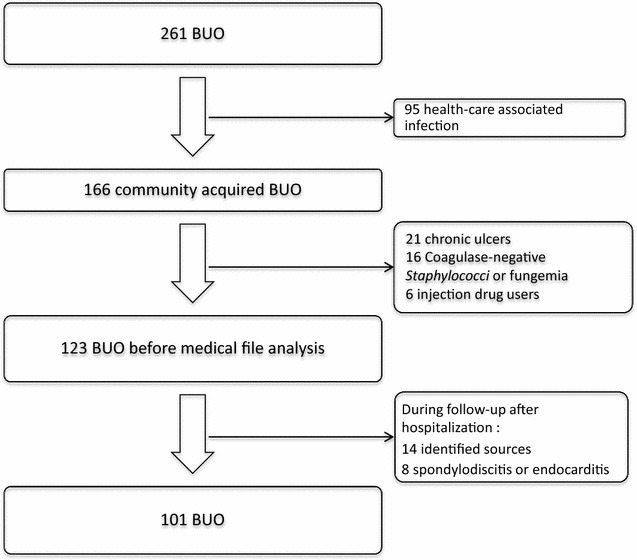
Selection of the patients with a bacteremia of unknown origin (BUO)

Between July 2005 and April 2016 the medical dashboard recorded 13,576 hospitalizations. A bacteremia was detected among 1989 (15%) patients with 261/1989 (13%) classified in the group BUO. After applying the inclusion criteria to the 261 patients, 101 community-acquired BUO were retained (Fig. 1). The characteristics of the population studied are presented in Table 1. Thirty-one (31%) patients were immunocompromised: 12 with neoplasms (diagnosed during the last year), six with cirrhosis, six HIV-infected (two with CD4 T-cells under 200/mm^3^), three splenectomized patients, three with immunosuppressor drugs and 1 chronic renal insufficiency undergoing dialysis.

**Table 1:** Characteristics of the 101 patients with BUO

Clinical characteristics of patients	Values (%)
Age, years mean (SD)	71 (17)
Sex, men (%)	68 (67)
Comorbidities	
Cardiovascular	61 (60)
Immunosuppressed	31 (31)
Pulmonary	27 (27)
Diabetes mellitus	17 (17)
Dyslipidemia	14 (14)
Neurological	14 (14)
Medical device	17 (17)
Severe sepsis	52 (51)
Death	9 (9)
ICU transfer	12 (12)
Symptoms at admission	
Fever	99 (98)
Isolated fever	22 (22)
Dyspnea	14 (14)
Fatigue	13 (13)
Diarrhea/vomiting	13 (13)
Confusion	13 (13)
Loss of consciousness	11 (11)
Back pain	8 (8)
Arthralgia/myalgia	6 (6)
Fall	6 (6)
Chest pain	4
Abdominal pain	3
Neurological deficit	2
Jaundice	1
Cough	1
Headache	1

An antibiotic treatment had been prescribed the month preceding the BUO in 11/101 (11%) patients. Fever was detected at day 1 in 99/101 (98%) patients. The median duration of symptoms leading to the emergency room visit was 3 days. Severe sepsis was identified in 52/101 (51%) patients. ICU management was required for 12/101 (12%) patients. CRP and PCT values at admission were available in 98/101 (97%) and 47/101 (46%) of the cases respectively. The CRP value was below 20 mg/L in 17/98 (17%) patients and PCT value below 0.5 ng/L in 10/47 (21%) patients. A thoracic and abdominal CT-Scan was performed in 69/101 (68%) patients, colonoscopy in 37/101 (36%), TTE in 56/101 (55%) patients and TOE in 29/101 (28%).

Empirical antibiotic therapy was started in 55/101 (54%) patients and was inadequate in 9/55 (16%) cases. Empirical antibiotic administration was significantly associated with occurrence of organ dysfunction (70 vs 39%, p = 0.002). Unfavorable outcome occurred in 17/101 (17%) patients. In case of inadequate antibiotic there was a trend toward unfavorable outcome (29 vs 13%, p = 0.16). *S. aureus* and diarrhea and/or vomiting at admission were associated with unfavorable outcome in multivariable analysis (Table 2). The in-hospital mortality rate was 9% (9/101) during community-acquired BUO and 6.4% (862/13,475) for all the other patients hospitalized during the study period. Among the nine deaths three septic shocks occurred. The median follow-up duration was 18 months and 13/101 (13%) of patients were lost to follow up. Recurrence of BUO was observed in 10/101 (10%) patients including five with bacteria presenting the same antibiotype than the first episode.

**Table 2:** Risk factors for unfavourable outcome (death or ICU transfer) during in-hospital care

Variables	Unfavourable outcome n = 17 (17)	Favourable outcome n = 84 (83)	p	OR [95% CI]
Age, years (mean)	70	74	0.335	
Sex (male)	9 (53)	59 (70)	0.165	
Comorbidities				
Cardiovascular	11 (65)	50 (60)	0.690	
Immunosuppressed	3 (18)	28 (33)	0.232	
Pulmonary	6 (35)	21 (25)	0.381	
Diabetes mellitus	3 (18)	14 (16)	0.921	
Dyslipidemia	4 (23)	10 (12)	0.205	
Neurological	3 (18)	11 (13)	0.620	
Medical device	5 (30)	13 (15)	0.170	
Symptoms at admission				
Dyspnea	2 (12)	11 (13)	0.881	
Diarrhea/vomiting	5 (30)	7 (8)	0.014	2.47 [1.62–26.78]
Confusion	2 (12)	11 (13)	0.881	
Loss of consciousness	4 (23)	8 (9)	0.103	
Bacteria				
*Enterobacteriaceae*	6 (35)	37 (44)	0.505	
*Staphylococcus aureus*	5 (29)	6 (7)	0.007	2.64 [1.42–22.17]
*Streptococcus* spp.	5 (29)	33 (39)	0.443	
Polymicrobial	2 (12)	9 (11)	0.899	

Univariate and multivariate analysis

At least two blood culture bottles were positive for 94/101 (92%) BUO, eight patients had only one positive bottle with the following bacteria: 2 *Streptococcus gallolyticus*, 1 *Streptococcus oralis,* 1 *Escherichia coli,* 1 *Enterococcus faecalis,* 1 *Staphylococcus aureus* and 1 *Citrobacter amalonaticus*. The 115 bacteria involved in the BUO are presented in Table 3, 11/101 (11%) BUO were polymicrobial. The main species retrieved were *E. coli* 25/115 (22%), group D *Streptococci* 12/115 (10%), viridans *Streptococci* 12/115 (10%) and *S. aureus* 11/115 (9%). AMC/GM displayed a higher rate of effectiveness compared to 3GC/GM: 100/101 (99%) vs 94/101 (93%) (p = 0.04). In one patient a strain of *E. faecium* with modification of penicillin-binding protein 5 impaired both associations. *Bacteroides* spp. (lack of aminoglycoside uptake + class A β-lactamase) in three patients and *E. faecalis* (intrinsic resistance to cephalosporins and aminoglycoside) in three patients impaired only 3GC/GM. All the natural or acquired resistance mechanisms of the bacteria to one of the drug of the associations are presented in Table 3.

**Table 3:** Bacteria isolated and associated resistance to amoxicillin-clavulanic acid, gentamicin and 3rd generation cephalosporin

Bacteria	n	Natural resistance	n	Acquired resistance	n
*Enterobacteriaceae*					
*Escherichia coli*	25			High-level penicillinase (AMC-R)	3
*Klebsiella pneumoniae*	9			Inhibitor-resistant TEM (AMC-R)	2
*Enterobacter* spp.	5	ampC chromosomal inducible cephalosporinase (AMC-R^a^)	5	Cephalosporinase hyperproduction (AMC-R and 3GC-R^b^)	3
*Proteus mirabilis*	3			ESBL (AMC-R and 3GC-R)	1
Others	5				
Non-*Enterobacteriaceae* Gram-negative bacteria					
*Pseudomonas aeruginosa*	2	ampC chromosomal Inducible and low permeability (AMC-R and 3GC-R)	2		
Others	3				
*Streptococcus* spp.					
Group D *Streptococci*	12				
Viridans *Streptococci*	12				
*Streptococcus milleri* group	4				
*Streptococcus pyogenes*	3				
*Streptococcus agalactiae*	3				
*Streptococcus pneumoniae*	2				
Group C *Streptococci*	2				
*Enterococcus* spp.					
*Enterococcus faecalis*	3	PBP5 (3GC-R)	4		
*Enterococcus faecium*	1			High-level PBP5 (AMC-R)	1
*Staphylococcus aureus*	11			PBP2A (AMC-R and 3GC-R)	1
				APH 2^′′^-AAC 6^′^ (GM-R^c^)	2
Other Gram-positive					
*Gemella haemolysans*	1				
*Listeria monocytogenes*	2	PBP3 (3GC-R)	2		
Anaerobes		Lack of drug uptake (GM-R)	7		
*Bacteroides* spp.	4	Class A β-lactamase (3GC-R)	4		
*Fusobacterium nucleatum*	1				
*Parvimonas micra*	1				
*Eubacterium* spp.	1				

^a^Mechanism resulting in resistance to amoxicillin–clavulanic acid

^b^Mechanism resulting in resistance to ceftriaxone

^c^Mechanism resulting in resistance to gentamicin

## Discussion

This study conducted in one French University Hospital shows that community BUO mainly involves old male patients, with half of them presenting at least one organ dysfunction and requiring ICU admission in 12% of the cases. Isolated fever with a recent onset (less than 3 days) represents the primary clinical feature for 22% of the patients whereas symptoms leading to emergency room visit vary widely for the rest of the patients. *S. aureus* BUO was associated to unfavourable outcome. Based on the analysis of the antibiotic susceptibility tests AMC + GM provided a higher rate of adequacy than 3GC + GM when used empirically.

The dashboard of our ward classifies infective endocarditis and vertebral osteomyelitis in other groups. Unfortunately in those groups i.e. bone and joint infections and vascular infections, the portal of entry is not recorded. So a number of BUO with vascular or bone secondary focus has not been included thus representing a selection bias. In the same way exclusion of chronic cutaneous conditions could have lead to selection bias. Bacteriological analysis of samples collected on chronic cutaneous conditions as pressure ulcers is often polymicrobial and variable over time. Deep tissue biopsies are not routinely performed for this purpose so the exclusion of a cutaneous primary focus is never obvious. Since the most frequent clinical presentation of coagulase-negative *Staphylococci* is device-associated health care-associated infections [12] BUO with those bacteria had been excluded from our work focusing on community-acquired infection. The choice of the two beta-lactams studied associated to an aminoglycoside results from the research for a spectrum broad enough but with consideration for appropriate antibiotic use and ecologic impact.

In our dashboard, BUO corresponds to 13% of all the bacteremia. This result is close to the rates reported in a Spanish Emergency Department (12%) [13] and in a Spanish tertiary care center (16%) [5]. During the challenging management of BUO biomarkers may be helpful to the clinician. However, in our study, with a cut-off value of 0.5 ng/L the PCT yield a 21% rate of false negative. PCT is usually recognized as a biomarker able to rule out a bacteremia in patients presenting with fever in the emergency department [14]. In daily clinical practice a CRP value below 20 mg/L is usually considered not to be associated with a bacterial infection [15]. Compared to PCT, this cut-off provides a lower rate of false negative: 17%.

Bacterial epidemiology for *S. aureus*, *Enterobacteriaceae* and polymicrobial infections among the BUO are consistent with the few previously published data [5, 13, 16] while viridans *Streptococci*, *Enterococci* and group D *Streptococci* are more frequent in our study.

The high rate of patients presenting with at least one organ failure (51%) together with the 29% increase of the in-hospital mortality rate compare to the other patients hospitalized during the study period underscore the need to identify the best-suited empirical treatment. As in former studies, the definition of appropriate antibiotic used in our study considers monotherapy of aminoglycoside as efficient [17]. Compared to 3GC + GM, the use of AMC + GM enabled appropriate treatment in case of infection due to *Bacteroides* spp. and *E. faecalis*. ECDC antimicrobial resistance surveillance report [18] is the only tool providing resistance rate to antibiotic associations such as cephalosporins + aminoglycosides at an international level. Nonetheless those results include both community and health-care acquired infections and do not enable an assessment of our therapeutic strategy.

The fact that diarrhea and/or vomiting at admission is associated to unfavourable outcome remains unexplained. Similar to prior findings during BUO, *S. aureus* is associated with unfavourable outcome [5]. The use of beta-lactam/beta-lactamase inhibitor association for empirical treatment f *S. aureus* bacteremia has been associated with increased mortality rate compared to oxacillin or cefazolin [19]. Nevertheless in this study we propose the systematic use of a combination with an aminoglycoside known to significantly impact the course of *S. aureus* bacteremia [20].

Sepsis of unknown origin refers to a clinical situation encountered in the emergency room or just after ICU admission which includes: future BUO, sepsis with a primary source which will be documented in the following hours because of changes in clinical examination or results of paraclinical exams, sepsis which will never be documented and also non-infectious aetiologies [21]. So BUO represents only a subgroup of sepsis of unknown origin defined a posteriori on the basis of bloodcultures. Then our results regarding AMC + GM cannot be entirely and directly applied to sepsis of unknown origin. Our work based on patient hospitalized in an ID department aimed to use microbiological data of BUO as a surrogate marker to guide antimicrobial treatment during sepsis of unknown origin. As all surrogate markers it presents necessarily some drawbacks but to our knowledge no other work has intended to use a different one.

## Conclusion

When considering an empirical antibiotic treatment for community-acquired sepsis of unknown origin, microbiological features of BUO presenting half of the time with organ failure has to be integrated in the decision process. With our results, AMC + GM should be considered.

## References

[1] Viscoli C (2016). Bloodstream infections: the peak of the iceberg. Virulence.

[2] Weinstein MP, Towns ML, Quartey SM (1997). The clinical significance of positive blood cultures in the 1990s: a prospective comprehensive evaluation of the microbiology, epidemiology, and outcome of bacteremia and fungemia in adults. Clin Infect Dis.

[3] Renaud B, Brun-Buisson C, Group IC-BS (2001). Outcomes of primary and catheter-related bacteremia. A cohort and case-control study in critically ill patients. Am J Respir Crit Care Med.

[4] Valles J, Rello J, Ochagavia A, Garnacho J, Alcala MA (2003). Community-acquired bloodstream infection in critically ill adult patients: impact of shock and inappropriate antibiotic therapy on survival. Chest.

[5] Hernandez C, Cobos-Trigueros N, Feher C (2014). Community-onset bacteraemia of unknown origin: clinical characteristics, epidemiology and outcome. Eur J Clin Microbiol Infect Dis.

[6] Pedersen G, Schonheyder HC, Sorensen HT (2003). Source of infection and other factors associated with case fatality in community-acquired bacteremia—a Danish population-based cohort study from 1992 to 1997. Clin Microbiol Infect.

[7] Adrie C, Garrouste-Orgeas M, Ibn Essaied W (2017). Attributable mortality of ICU-acquired bloodstream infections: impact of the source, causative micro-organism, resistance profile and antimicrobial therapy. J Infect.

[8] Garnacho-Montero J, Gutierrez-Pizarraya A, Escoresca-Ortega A, Fernandez-Delgado E, Lopez-Sanchez JM (2015). Adequate antibiotic therapy prior to ICU admission in patients with severe sepsis and septic shock reduces hospital mortality. Crit Care.

[9] Canton R, Novais A, Valverde A (2008). Prevalence and spread of extended-spectrum beta-lactamase-producing Enterobacteriaceae in Europe. Clin Microbiol Infect.

[10] Roger PM, Farhad R, Leroux S (2008). Gestion de services, tarification à l’activité, recherche clinique et évaluation des pratiques professionnelles: un même outil informatique. Med Mal Infect.

[11] Comité de l’antibiogramme de la société Française de Microbiologie. http://www.sfm-microbiologie.org/page/page/showpage/page_id/81.html.

[12] Becker K, Heilmann C, Peters G (2014). Coagulase-negative staphylococci. Clin Microbiol Rev.

[13] Ortega M, Almela M, Martinez JA (2007). Epidemiology and outcome of primary community-acquired bacteremia in adult patients. Eur J Clin Microbiol Infect Dis.

[14] Chirouze C, Schuhmacher H, Rabaud C (2002). Low serum procalcitonin level accurately predicts the absence of bacteremia in adult patients with acute fever. Clin Infect Dis.

[15] Knudtzen FC, Nielsen SL, Gradel KO (2014). Characteristics of patients with community-acquired bacteremia who have low levels of C-reactive protein (</=20 mg/L). J Infect.

[16] Leibovici L, Konisberger H, Pitlik SD, Samra Z, Drucker M (1992). Bacteremia and fungemia of unknown origin in adults. Clin Infect Dis.

[17] Marquet K, Liesenborgs A, Bergs J, Vleugels A, Claes N (2015). Incidence and outcome of inappropriate in-hospital empiric antibiotics for severe infection: a systematic review and meta-analysis. Crit Care.

[18] European Centre for Disease Prevention and Control (ECDC). Antimicrobial resistance surveillance in Europe 2014. Annual Report of the European Antimicrobial Resistance Surveillance Network (EARS-Net). Stockholm: ECDC; 2015. http://ecdc.europa.eu/en/publications/Publications/antimicrobial-resistance-europe-2014pdf.

[19] Paul M, Zemer-Wassercug N, Talker O (2011). Are all beta-lactams similarly effective in the treatment of methicillin-sensitive *Staphylococcus aureus* bacteraemia?. Clin Microbiol Infect.

[20] Lemonovich TL, Haynes K, Lautenbach E, Amorosa VK (2011). Combination therapy with an aminoglycoside for Staphylococcus aureus endocarditis and/or persistent bacteremia is associated with a decreased rate of recurrent bacteremia: a cohort study. Infection.

[21] Contou D, Roux D, Jochmans S (2016). Septic shock with no diagnosis at 24 hours: a pragmatic multicenter prospective cohort study. Crit Care.

